# Inflammatory cytokines-stimulated human muscle stem cells ameliorate ulcerative colitis via the IDO-TSG6 axis

**DOI:** 10.1186/s13287-020-02118-3

**Published:** 2021-01-09

**Authors:** Shengchao Zhang, Jiankai Fang, Zhanhong Liu, Pengbo Hou, Lijuan Cao, Yuyan Zhang, Rui Liu, Yanan Li, Qianwen Shang, Yongjing Chen, Chao Feng, Guan Wang, Gerry Melino, Ying Wang, Changshun Shao, Yufang Shi

**Affiliations:** 1 The First Affiliated Hospital of Soochow University, Institutes for Translational Medicine, State Key Laboratory of Radiation Medicine and Protection, Key Laboratory of Stem Cells and Medical Biomaterials of Jiangsu Province, Medical College of Soochow University, 199 Renai Road, Suzhou, 215123 Jiangsu People’s Republic of China; 2 Department of Experimental Medicine and Biochemical Sciences, University of Rome Tor Vergata, Rome, Italy; 3 Shanghai Institute of Nutrition and Health, Shanghai Institutes for Biological Sciences, Chinese Academy of Sciences, Shanghai, People’s Republic of China; 4 Institutes for Translational Medicine, State Key Laboratory of Radiation Medicine and Protection, Medical College of Soochow University, Suzhou, Jiangsu People’s Republic of China

**Keywords:** Human muscle stem cells, Inflammatory bowel disease, Indoleamine 2,3-dioxygenase, TNF-stimulated gene 6, Kynurenine, Kynurenic acid, Aryl hydrocarbon receptor

## Abstract

**Background:**

Muscle stem cells (MuSCs) are absolutely required for the formation, repair, and regeneration of skeletal muscle tissue. Increasing evidence demonstrated that tissue stem cells, especially mesenchymal stem cells (MSCs), can exert therapeutic effects on various degenerative and inflammatory disorders based on their immunoregulatory properties. Human mesenchymal stem cells (hMSCs) treated with interferon-γ (IFN-γ) and tumor necrosis factor-α (TNF-α) were reported to possess anti-inflammatory functions by producing TNF-stimulated gene 6 (TSG-6). However, whether human muscle stem cells (hMuSCs) also possess TSG-6 mediated anti-inflammatory functions has not been explored.

**Methods:**

The ulcerative colitis mouse model was established by subjecting mice to dextran sulfate sodium (DSS) in drinking water for 7 days. hMuSCs were pretreated with IFN-γ and TNF-α for 48 h and were then transplanted intravenously at day 2 of DSS administration. Body weights were monitored daily. Indoleamine 2,3-dioxygenase (IDO) and TSG-6 in hMuSCs were knocked down with short hairpin RNA (shRNA) and small interfering RNA (siRNA), respectively. Colon tissues were collected for length measurement and histopathological examination. The serum level of IL-6 in mice was measured by enzyme-linked immunosorbent assay (ELISA). Real-time PCR and Western blot analysis were performed to evaluate gene expression.

**Results:**

hMuSCs treated with inflammatory factors significantly ameliorated inflammatory bowel disease (IBD) symptoms. IDO and TSG-6 were greatly upregulated and required for the beneficial effects of hMuSCs on IBD. Mechanistically, the tryptophan metabolites, kynurenine (KYN) or kynurenic acid (KYNA) produced by IDO, augmented the expression of TSG-6 through activating their common receptor aryl hydrocarbon receptor (AHR).

**Conclusion:**

Inflammatory cytokines-treated hMuSCs can alleviate DSS-induced colitis through IDO-mediated TSG-6 production.

**Supplementary Information:**

The online version contains supplementary material available at 10.1186/s13287-020-02118-3.

## Background

As the most abundant tissue of the human body, skeletal muscle comprises approximately 40% of total body mass. It fulfills multiple functions in the body such as postural support, voluntary locomotion, breathing, and endocrine and paracrine production, and regulates thermogenesis and systemic metabolism [1, 2]. Skeletal muscle possesses a remarkable regenerative capacity, which is attributed to a population of resident adult stem cells, called muscle stem cells (MuSCs), located in between the plasma membrane of the muscle fiber and the surrounding basal lamina [3]. MuSCs are quiescent under homeostatic conditions in adults. Following injury and disease, MuSCs are activated to enter the cell cycle, proliferate, differentiate, and fuse to form new muscle fibers, which replace damaged and necrotic muscle tissues [4]. This process is also termed as “cell replacement”.

Beyond the physical replacement of damaged cells, inflammation-licensed stem cells also orchestrate the remodeling of tissue microenvironments by producing immunoregulatory factors as well as growth factors that dictate the functions of other resident progenitor cells, which is termed as “cell empowerment.” For example, mesenchymal stem cells (MSCs) can facilitate the repair and regeneration of damaged tissues through suppressing innate and adaptive immune responses [5–8]. However, their immunosuppressive properties are not constitutive, but need to be licensed or elicited by inflammatory cytokines, specifically interferon-γ (IFN-γ) in combination with tumor necrosis factor-α (TNF-α) or interleukin-1β (IL-1β). Upon tissue damage, activated MSCs produce various anti-inflammatory mediators, such as indoleamine 2,3-dioxygenase (IDO), TNF-stimulated gene 6 (TSG-6), and transforming growth factor-β (TGF-β), which modulate the progression of inflammation, promote angiogenesis, remodel extracellular matrix (ECM), and facilitate the proliferation and differentiation of tissue progenitor cells [5, 8–11].

IDO, an enzyme determining the first and rate-limiting step of tryptophan degradation along the kynurenine pathway, plays a vital role in the immunomodulatory functions of hMSCs [12–14]. Notably, IDO and tryptophan metabolites, such as kynurenine (KYN), kynurenic acid (KYNA), and 3-hydroxyanthranilic, have been documented to modulate the functions of immune cells and regulate the expressions of inflammation-associated genes [15–18]. In addition, TSG-6, a 30-kDa secreted glycoprotein, has been shown to have strong anti-inflammatory properties in acute lung injury, peritonitis, and rheumatoid arthritis [19–21]. Inflammatory bowel disease (IBD), encompassing Crohn’s disease (CD) and ulcerative colitis (UC), is a chronic relapsing-remitting inflammatory disease of the gastrointestinal tract [22]. While CD may involve any area in the gastrointestinal tract and presents with transmural inflammation, fistula, or combination phenotype, UC is characterized by mucosal inflammation and limited to the colon [19]. Interestingly, the therapeutic effect of MSCs on colitis was shown to be mediated by TSG-6 [23]. Previous research has demonstrated that TSG-6 inhibits neutrophil migration to inflammatory sites [24]. Moreover, TSG-6 can convey ECM mediating cellular signaling by interacting with glycosaminoglycans (GAGs) such as hyaluronan (HA), heparan sulfate (HS), and heparin [20]. Additionally, TSG-6 released from hMSCs ameliorates DSS-induced colitis through inducing M2 macrophage polarization [21, 25].

In recent years, human muscle stem cells (hMuSCs) have garnered substantial interest due to their critical roles in aging, exercise, and neuromuscular diseases [1, 26, 27]. However, few studies have assessed the immunoregulatory functions of hMuSCs in inflammatory disorders. Our previous study demonstrated that mouse MuSCs (mMuSCs) can confer maturing macrophages anti-inflammatory properties through insulin-like growth factor-2 (IGF-2) to alleviate IBD [28]. Notably, the counterparts of IDO and TSG-6 are not expressed in the corresponding mouse cells [13]; thus, the role of IDO-TSG-6 axis in immunoregulation can be better studied with human tissue stem cells.

In this study, we found that hMuSCs exerted anti-inflammatory effects in IBD mice through releasing TSG-6, and TSG-6 production was regulated by IDO. Further analysis demonstrated that tryptophan metabolites, KYN or KYNA produced by IDO, augmented TSG-6 expression through activating aryl hydrocarbon receptor (AHR). Our study reveals a novel TSG-6-dependent immunosuppressive function of hMuSCs, which could expand the application of hMuSC-based cell therapy to inflammatory diseases.

## Materials and methods

### Isolation, expansion, and differentiation of hMuSCs

Surgical specimens of human skeletal muscle were isolated in accordance with the Ethics Committee of the First Affiliated Hospital of Soochow University. All specimens were debrided tissues from patients with orthopedic trauma and obtained with informed consent of the patients. Specimens of gastrocnemius were used in all experiments. Human skeletal muscle was obtained and stored in a clean 50-ml centrifuge tube containing medium (DMEM low), for processing within 3 h of collection. hMuSCs were isolated and cultured as previously described [29, 30]. Briefly, human skeletal muscle tissue was first rapidly minced and incubated in 50 ml centrifuge tube containing collagenase II (750 U/ml, Gibco, Carlsbad, CA, USA) at 37 °C for 60 min in a shaker. The digested muscle was washed once with cold wash buffer (DMEM low supplemented with 10% horse serum (Gibco, MA, USA) and 1% penicillin-streptomycin). Then, collagenase II (100 U/ml) and dispase (11 U/ml, Gibco) solution were added at 50 ml centrifuge tube to incubation at 37 °C for 30 min in a shaker. The digested tissues were filtered with 40-μm nylon cell strainer to obtain single cells. These cells were stained with anti-CD31-PE, anti-CD34-PE, anti-CD45-PE, anti-CD29-APC, anti-CD56-V450, and anti-EGFR-V450 (both from BioLegend, San Diego, CA, USA) for 45 min at 4 °C. All antibodies were used at ~ 1 μg per 10^7^ cells. The CD31^−^CD34^−^CD45^−^CD29^+^CD56^+^EGFR^+^ hMuSCs were obtained by fluorescence-activated cell sorter.

The sorted hMuSCs were cultured in myogenic growth medium containing 1:1 mixture of DMEM low medium:MCDB 131 medium, 20% fetal bovine serum (FBS), 1% penicillin-streptomycin (all from Gibco, MA, USA), 1% insulin-transferrin-selenium (ITS, Invitrogen, Carlsbad, CA, USA), and 10 μM p38 MAPK inhibitor (SB203580, Selleck, Houston, TX, USA), which functions to block the differentiation of hMuSCs and thus enables their self-renewal properties [30]. Cell plates and slides were precoated with ECM (Sigma-Aldrich, St Louis, MO, USA). Cultured hMuSCs can differentiate into myofibers in myogenic cell differentiation medium containing DMEM low with 5% horse serum (Gibco, MA, USA) for 3 days. All details regarding the characterization of cultured hMuSCs were shown in Supplementary Figure 1.

### Transfection of hMuSCs with shRNA/siRNA

For knockdown of IDO, hMuSCs were transduced with IDO-targeting shRNA carried on a lentivirus vector (PGLV3/H1/GFP/Puro). Lentiviral shRNA was produced by co-transfection of the Trans-Lentiviral Packaging Mix with a shRNA transfer vector into HEK 293T packaging cells. Supernatants containing either the lentivirus expressing the IDO shRNA or the control shRNA were harvested 72 h after transfection. The lentiviruses were purified using ultracentrifugation, and the titers of the lentiviruses were determined. IDO shRNA sequence was 5′-GCGCTGTTGGAAATAGCTTCT-3′. The sequence of control shRNA was 5′-TTCTCCGAACGTGTCACGT-3′. hMuSCs were incubated with lentivirus and 5 μg/ml Polybrene for 24 h. Puromycin (5 μg/ml, Gibco) was added into cultured medium to select transduced cells.

For knockdown of TSG-6, hMuSCs were transfected with TSG-6 siRNA or control siRNA (GenePharma) with Lipofectamine RNAiMAX (Invitrogen) according to the instructions of manufacturer. After 24 h, cells were treated with indicated inflammatory factors. The efficiency of knockdown was detected by real-time PCR and ELISA. TSG-6 siRNA sequence was 5′-GGGAAGAUACUGUGGAGAUTT-3′ and 5′-AUCUCCACAGUAUCUUCCCTT-3′.

### DSS-induced colitis and experimental therapies

C57BL/6 mice (6–8 weeks old) were purchased from Vital River Laboratory Animal Technology Co., Ltd. (Beijing, China), and maintained under specific pathogen-free conditions of the Laboratory Animal Center of Soochow University. Male mice were used in our study. All animal experiment protocols were approved by the Institutional Animal Care and Use Committee of Soochow University. Colitis was induced by 4% DSS (MP Biomedicals, Solon, OH, USA) in drinking water ad libitum from day 0 to day 7. DSS solution was refreshed every 2 days. The mice receiving normal drinking water were used as healthy controls. On day 2, hMuSCs or hMuSCs pretreated with IFN-γ and TNF-α for 48 h were harvested and washed three times with PBS, and then, these cells (2.5 × 10^5^ cells in 200 μl PBS) were administered intravenously into mice and PBS was injected as vehicle control; the recombination human TSG-6 protein (10 μg rhTSG-6 in 200 μl PBS, R&D Systems, Minneapolis, MN) was administered through intraperitoneal injection from day 2 to day 7. Body weight of each mouse was recorded every day. All mice were sacrificed on day 7; the serum and colon samples of mice were obtained for further experiments.

### Histological analysis of colons

Colon tissues were fixed in 4% paraformaldehyde for 48 h, dehydrated by alcohol concentration gradient, and embedded in paraffin, and then, the samples were cut into 3-μm-thick sections, which were stained with hematoxylin and eosin (H&E). The severity of IBD symptoms was evaluated by scoring the extent of bowel wall thickening (grades, 0–3: 0, none; 1, mucosa; 2, mucosa and submucosa; 3, transmural), the damage of crypt (grades, 0–3: 0, none; 1, loss of goblet cells; 2, only surface epithelium intact; 3, loss of entire crypt and epithelium), and the infiltration of inflammatory cells (grades, 0–2: 0, none; 1, mild to moderate; 2, severe).

### Real-time PCR

Total RNA was extracted using RNAprep Pure Cell Kit (Feijie Biotech, Shanghai, China), and reverse-transcribed into cDNA with PrimeScript™ RT Master Mix (TaKaRa Biotech, Dalian, China). The levels of mRNA expression were analyzed by QuantStudio™ 6 Flex System according to the manufacturer’s instructions. The total reaction volume of 10 μl was comprised of 1 ng cDNA, 3 μl DNAase/RNAse-free water (TaKaRa Biotech, China), 1 μl primers (GENEWIZ, Suzhou, China), and 5 μl SYBR qPCR SuperMix plus (with ROX) (Novoprotein, Shanghai, China). The real-time PCR conditions were as follows: pre-denaturation at 95 °C for 30 s, then followed by 40 cycles of denaturation at 95 °C for 5 s, annealing and extension at 60 °C for 30 s. The total amount of mRNA was compared with endogenous β-actin mRNA. Finally, the relative expression of mRNA was calculated using 2^–∆∆Ct^ method. Primer sequences were shown in Supplementary Table 1.

### Western blot analysis

Cells were digested and washed two times with PBS, and then lysed in RIPA lysis buffer containing phenylmethanesulfonyl fluoride (PMSF, Beyotime, Shanghai, China) for 30 min at 4 °C. The concentrations of protein were assayed by Pierce™ BCA Protein Assay Kit (ThermoFisher, Waltham, MA, USA). The protein samples were separated by 10% SDS-PAGE and transferred to PVDF membrane. The membrane was blocked by 5% BSA in PBS supplemented with 0.05% Tween 20 for 1 h and then incubated with IDO antibody (Abcam, Cambridge, UK) at 4 °C overnight. After washing three times with TBST (10 min each), the membrane was incubated with HRP-conjugated secondary antibody for 2 h at room temperature and washed three times again with TBST. The levels of proteins were detected through enhanced chemiluminescence (Beyotime).

### TSG-6 and IL-6 ELISA

The protein level of TSG-6 in the supernatant of hMuSCs treated with inflammatory cytokines was detected by ELISA (R&D Systems) as in reference [31]. For IL-6 analysis, the blood of mice was centrifuged at room temperature at 2500 rpm for 30 min, and then, the serum was collected into new 1.5 ml EP tubes. The content of IL-6 in serum was measured by Mouse IL-6 ELISA kit according to the manufacturer’s instructions (Beyotime).

### Statistical analysis

All data were shown as the mean ± standard error of the mean (SEM). For two-group comparison, two-tailed unpaired *t* tests were performed. For multiple group comparison, one-way analysis of variance test was performed. *P* values less than 0.05 were considered statistically significant.

## Results

### IDO is vital for the anti-inflammatory effects of hMuSCs on DSS-induced IBD

To investigate the immunoregulatory functions of hMuSCs in inflammatory diseases, we injected intravenously hMuSCs, untreated or stimulated with IFN-γ and TNF-α (I+T), into mice during IBD induction by DSS. The body weight loss in IBD mice was significantly mitigated by both types of hMuSCs compared with mice injected with PBS, though I+T conferred hMuSCs increased beneficial effects (Fig. S2a). Moreover, measurements of colon length in the I+T-hMuSC-treated IBD mice showed a similar trend (Fig. S2b). Histological examination showed that hMuSCs treated with I+T were more effective in alleviating bowel wall thickening, crypt damage, and the infiltration of inflammatory cells in colons (Fig. S2c). More importantly, the level of IL-6 in serum, an important indicator for inflammatory progression, was lowest in the I+T hMuSC group (Fig. S2d). These results indicated that while hMuSCs could exert anti-inflammatory effects on IBD, those stimulated by inflammatory cytokines acquired augmented potency. Thus, we choose to use hMuSCs pretreated with I+T in the subsequent experiments.

**Fig. 1:**
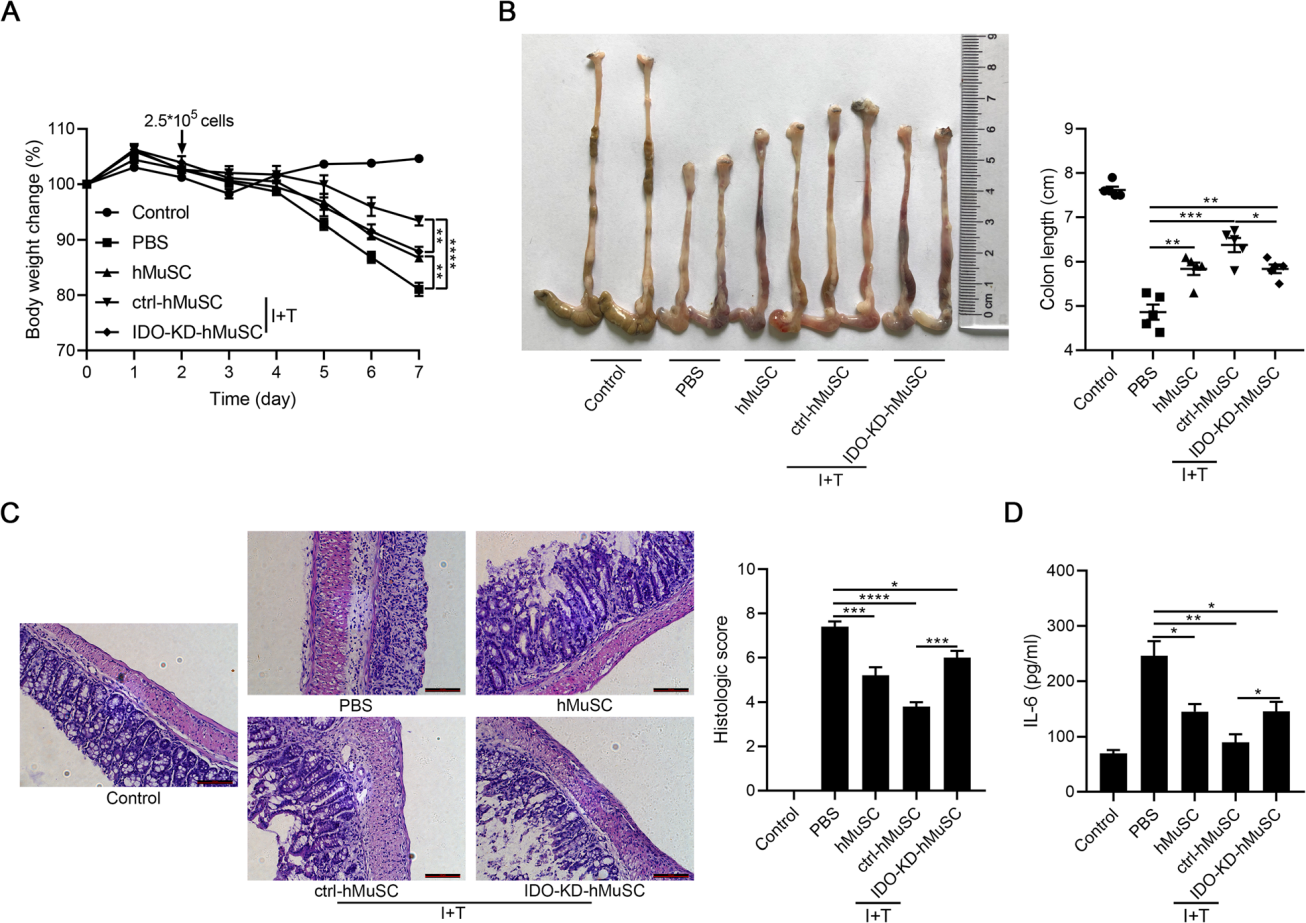
IDO knockdown partly impairs therapeutic effects of hMuSCs on IBD. **a** Mice with DSS-induced IBD were administered intravenously with PBS, hMuSCs, ctrl-hMuSCs, or IDO-KD-hMuSCs (2.5 × 10^5^ cells) on day 2. Ctrl-hMuSCs and IDO-KD-hMuSCs were pretreated with IFN-γ (10 ng/ml) and TNF-α (10 ng/ml) for 48 h. Changes in body weight during the entire experiment were shown as the percentage of the initial body weight on day 0. **b** Colon length of IBD mice was assessed. **c** H&E staining of the colon sections and histological scores were shown. **d** IL-6 in the serum of IBD mice was analyzed by ELISA. Five mice per group were used. Scale bars, 100 μm. Results were shown as mean ± SEM. *P < 0.05, **P < 0.01, ***P < 0.001, ****P < 0.0001

Given that IDO is the key molecule for the immunomodulatory capacity of hMSCs [12–14], we next determined whether IDO also mediates the immunomodulatory effects of hMuSCs. Hence, we established stable IDO knockdown cell line (IDO-KD-hMuSCs) using lentivirus transfection and injected intravenously IDO-KD-hMuSCs into IBD mice (Fig. S3). As expected, IDO insufficiency impaired therapeutic effects of hMuSCs on IBD mice (Fig. 1a–d). Thence, as in hMSCs, IDO also mediates the anti-inflammatory effects of hMuSCs conferred by inflammatory cytokines.

### hMuSCs alleviate IBD through IDO-dependent expression of TSG-6

**Fig. 2:**
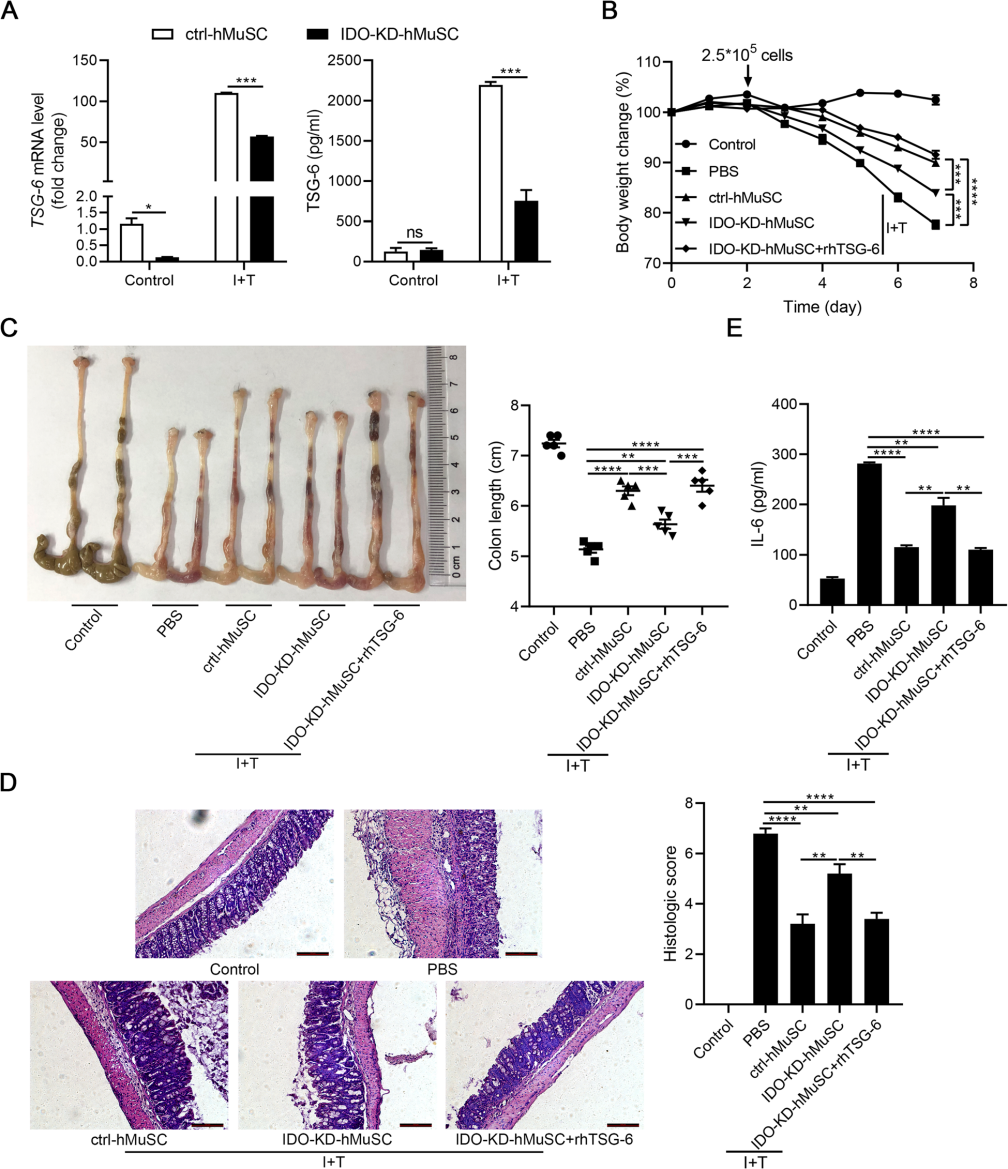
TSG-6 rescues the loss of therapeutic effects of IDO-KD-hMuSCs on IBD. **a** Ctrl-hMuSCs and IDO-KD-hMuSCs were treated with IFN-γ (10 ng/ml) and TNF-α (10 ng/ml) for 24 h, and the gene expression (left) and protein level (right) of TSG-6 were measured by real-time PCR and ELISA, respectively. **b** Ctrl-hMuSCs or IDO-KD-hMuSCs were pretreated with IFN-γ (10 ng/ml) and TNF-α (10 ng/ml) for 48 h, and then, these cells (2.5 × 10^5^ cells per mouse) were intravenously injected into IBD mice on day 2. The rhTSG-6 protein (2 μg per mouse) was administered intraperitoneally every day after cell administration. Changes in body weight during the entire experiment were shown as the percentage of the initial body weight on day 0. **c** Colon length of IBD mice was assessed. **d** H&E staining of the colon sections and histological scores were shown. **e** IL-6 in the serum of IBD mice was analyzed by ELISA. Five mice per group were used. Scale bars, 100 μm. Results were shown as mean ± SEM. *P < 0.05, **P < 0.01, ***P < 0.001, ****P < 0.0001; ns, no significance

We recently showed that the anti-inflammatory effects of hMSCs in an acute lung injury mouse model are exerted via IDO-TSG-6 axis [16]. TSG-6 is able to inhibit the infiltration of inflammatory cells, especially macrophages and neutrophils [24, 32]. Several studies also reported that TSG-6 ameliorates DSS-induced colitis through modulating the functions of immune cells in colons [21, 33]. Considering that DSS-induced IBD is mainly due to inflammatory damages inflicted by macrophages and neutrophils, we thus explored whether hMuSCs also ameliorated IBD through upregulating the IDO-TSG-6 axis. We found that IDO depletion led to a significant downregulation of TSG-6 (Fig. 2a). To verify that the therapeutic effects of IDO are mediated by TSG-6, IBD mice were treated with IDO-KD-hMuSCs combined with recombinant human TSG-6 protein (rhTSG-6). We found that the loss in the therapeutic effects of IDO-KD-hMuSCs was almost completely rescued by rhTSG-6 (Fig. 2b–e). Therefore, these results demonstrated that the anti-inflammatory effects of hMuSCs on IBD mice are dependent on IDO-regulated TSG-6.

**Fig. 3:**
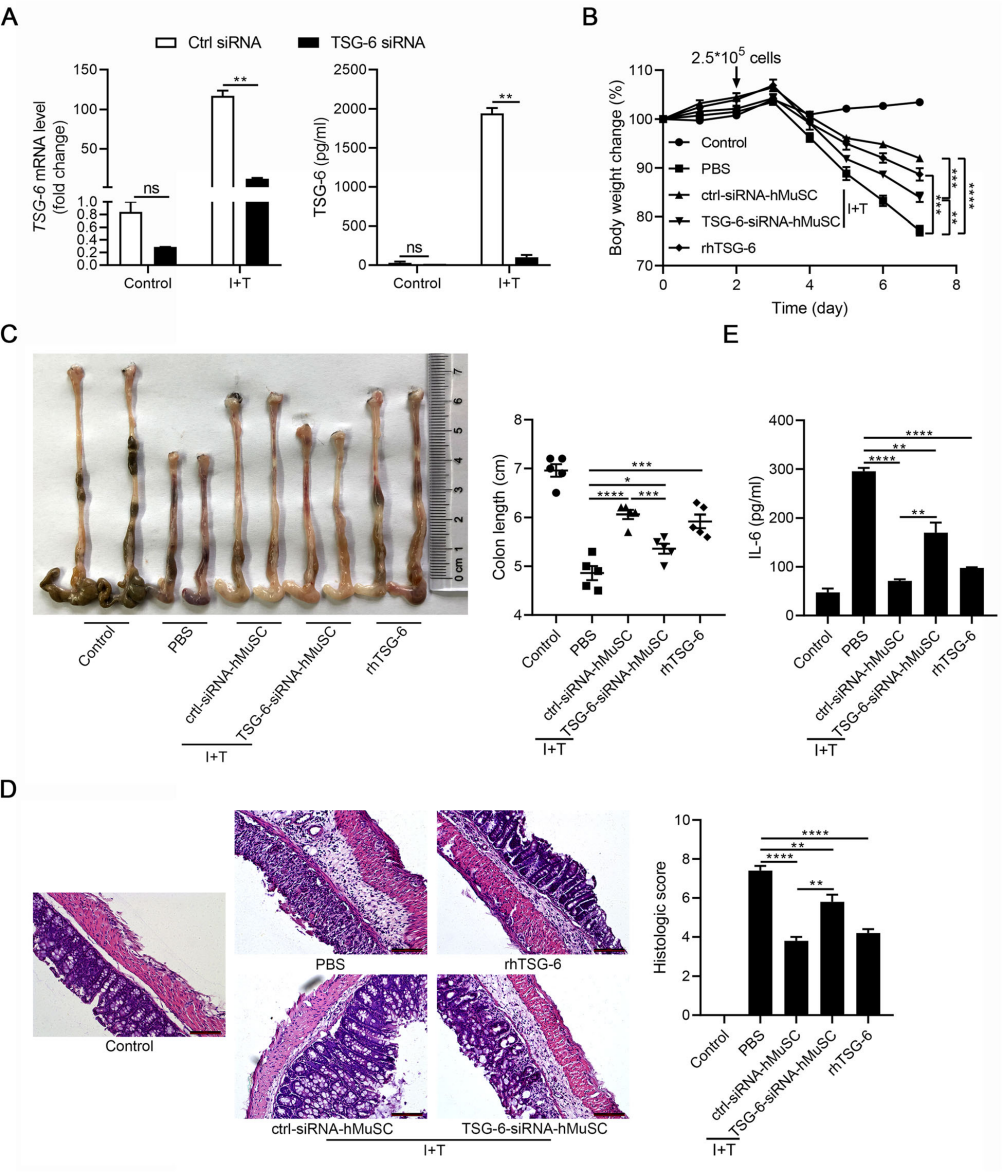
hMuSCs ameliorate IBD through secreting TSG-6. **a** hMuSCs transfected with control siRNA (ctrl-siRNA-hMuSCs) or TSG-6 siRNA (TSG-6-siRNA-hMuSCs) were pretreated with IFN-γ (10 ng/ml) and TNF-α (10 ng/ml) for 24 h to examine the expression of TSG-6 mRNA (left) and protein (right). **b** Ctrl-siRNA-hMuSCs and TSG-6-siRNA-hMuSCs were pretreated with IFN-γ (10 ng/ml) and TNF-α (10 ng/ml) for 48 h, and then, these cells (2.5 × 10^5^ cells per mouse) were intravenously injected into IBD mice on day 2. The rhTSG-6 protein (2 μg per mouse) was administered intraperitoneally from every day after cell administration. Changes in body weight during the entire experiment showed as the percentage of the initial body weight on day 0. **c** Colon length of IBD mice was assessed. **d** H&E staining of the colon sections and histological scores were shown. **e** IL-6 in the serum of IBD mice was analyzed by ELISA. Five mice per group were used. Scale bars, 100 μm. Results were shown as mean ± SEM. *P < 0.05, **P < 0.01, ***P < 0.001, ****P < 0.0001; ns, no significance

To further elucidate the functional role of TSG-6 in IBD, we transfected hMuSCs with siRNAs to knockdown TSG-6 (Fig. 3a, S3) and tested the anti-inflammatory effects of these cells on IBD mice. As expected, TSG-6 siRNA impaired the beneficial effects of hMuSCs, as reflected by changes in body weight, colon length, histologic score, and IL-6 serum level. Interestingly, IBD symptoms were greatly alleviated by exogenous rhTSG-6 (Fig. 3b–e). These data indicated that TSG-6 is critically required for hMuSCs to ameliorate IBD and support that the beneficial effects of hMuSCs on IBD are exerted through IDO-TSG-6 axis.

### KYN or KYNA regulates TSG-6 expression in hMuSCs

**Fig. 4:**
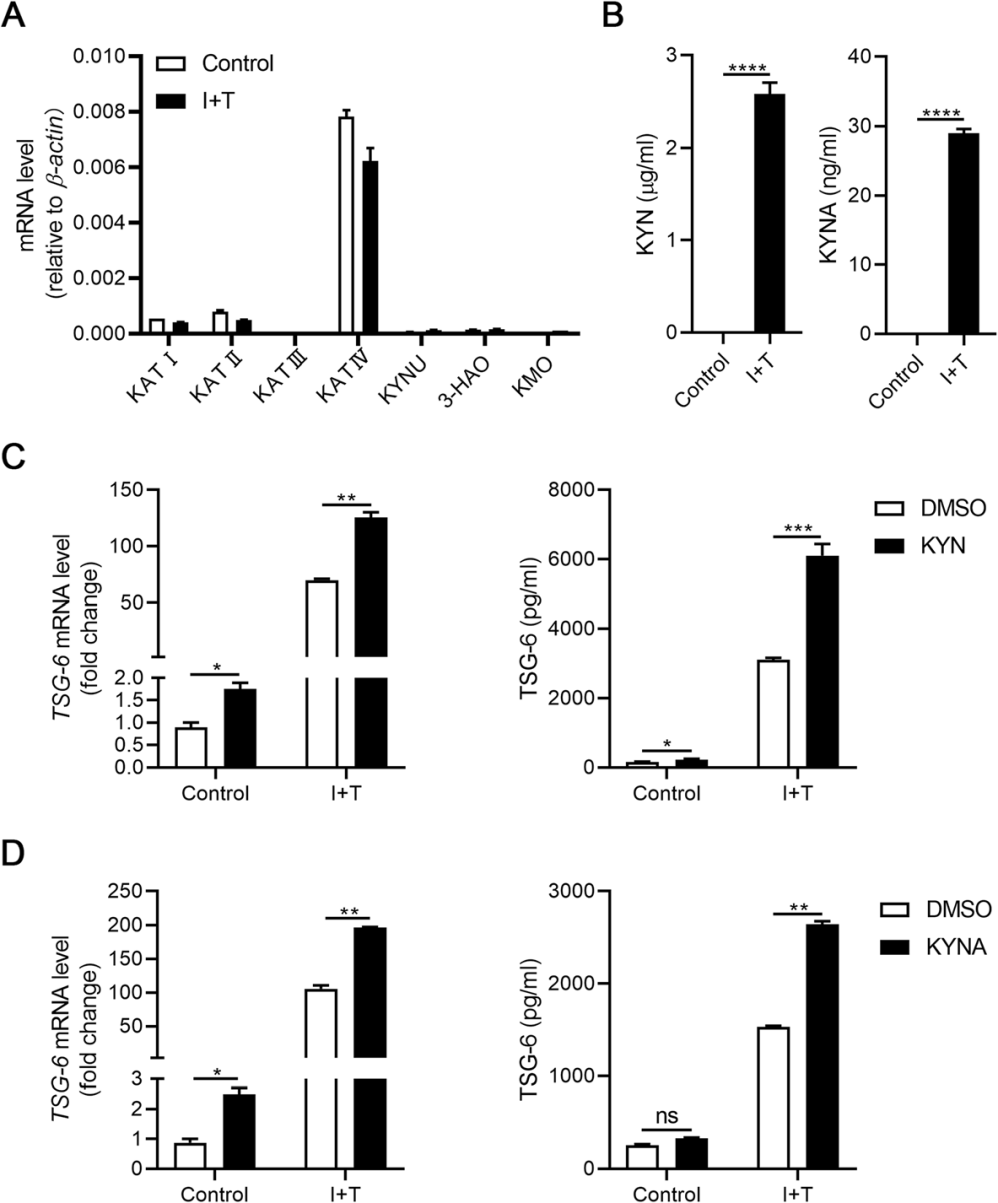
KYN or KYNA promotes TSG-6 production in hMuSCs. **a** hMuSCs were treated with IFN-γ (10 ng/ml) and TNF-α (10 ng/ml) for 24 h, and the expression levels of main enzymes in kynurenine pathway were examined by real-time PCR. **b** KYN and KYNA concentrations in the supernatant of hMuSCs treated with IFN-γ (10 ng/ml) and TNF-α (10 ng/ml) for 24 h were measured by HPLC-MS. **c**, **d** hMuSCs were treated with IFN-γ (1 ng/ml) and TNF-α (10 ng/ml) and were added DMSO, KYN (400 μM), or KYNA (200 μM). After 24 h, these cells were examined for the expression of TSG-6 mRNA (left) and protein (right). Results were shown as mean ± SEM. *P < 0.05, **P < 0.01, ***P < 0.001, ****P < 0.0001; ns, no significance

Recent studies have revealed that IDO can exert immunosuppressive functions either directly as signaling factor or by the generation of tryptophan metabolites, such as KYN, KYNA, and 3-hydroxyanthranilic acid [15–18]. To explore the underlying mechanism of IDO regulating TSG-6 expression, we examined the main enzymes of tryptophan metabolism in KYN pathway and found that among the four kynurenine aminotransferases (KAT), which catalyze the formation of KYNA from KYN, *KAT IV* has the highest expression in hMuSCs (Fig. 4a). Consistently, the contents of KYN and KYNA were greatly increased in the supernatant of hMuSC treated with I+T, as measured by high-performance liquid chromatography (HPLC), suggesting that KYN and KYNA are among the main tryptophan metabolites in hMuSCs (Fig. 4b). To determine whether these two metabolites could affect the expression of TSG-6, we treated hMuSCs with exogenous KYN or KYNA. While exogenous KYN or KYNA alone only minimally induced TSG-6 expression in naïve hMuSCs, and TSG-6 was greatly induced by I+T, KYN or KYNA could further increase the TSG-6 production (Fig. 4c, d). Taken together, these results confirmed that IDO can upregulate TSG-6 expression through tryptophan metabolites KYN or KYNA in inflammation-primed hMuSCs.

### Exogenous KYN or KYNA rescues the therapeutic effects of IDO-KD-hMuSCs through enhancing TSG-6 expression

**Fig. 5:**
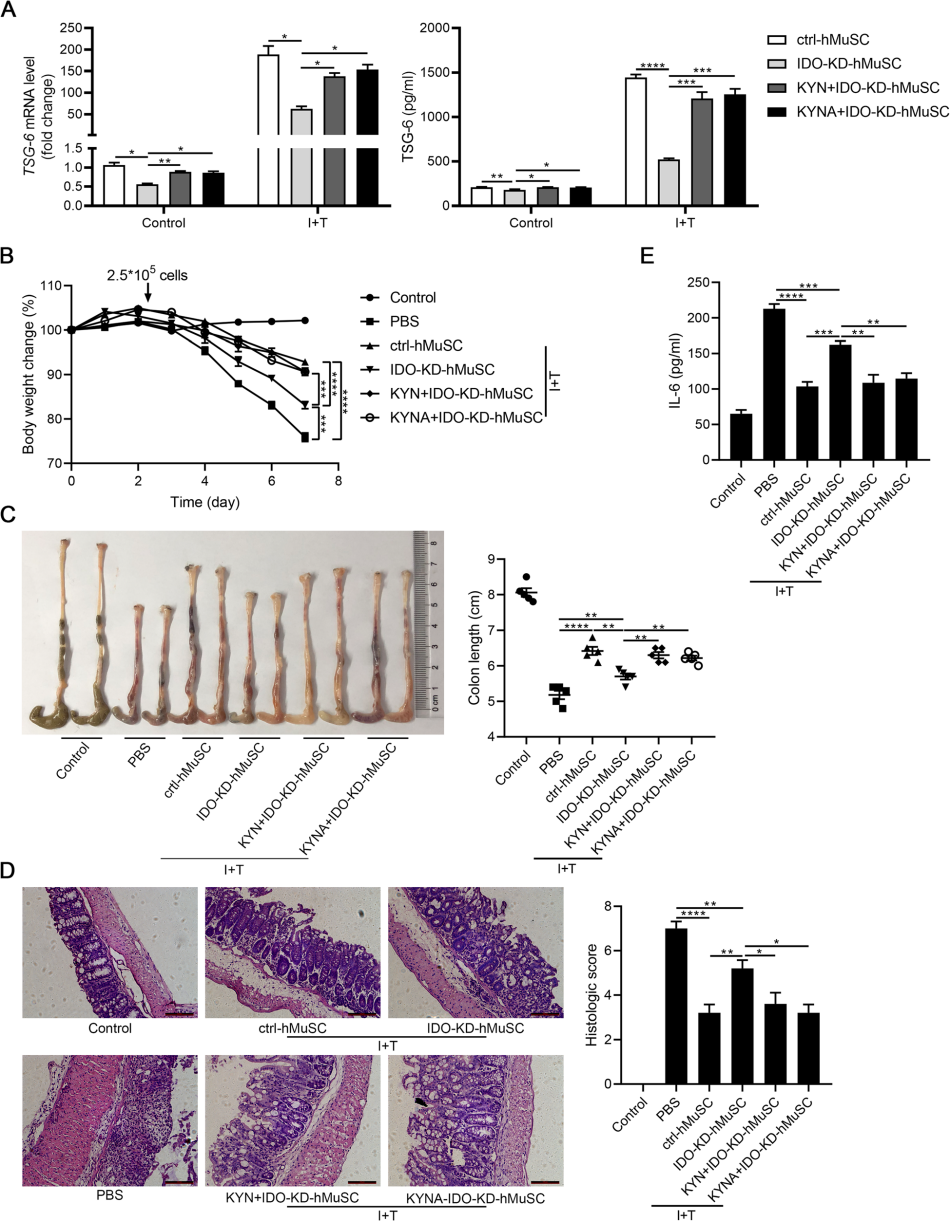
Exogenous KYN or KYNA rescues the loss of therapeutic effects of IDO-KD-hMuSCs on IBD through augmenting TSG-6 expression. **a** Ctrl-hMuSCs were treated with IFN-γ (10 ng/ml) and TNF-α (10 ng/ml), and IDO-KD-hMuSCs were treated with IFN-γ (10 ng/ml) and TNF-α (10 ng/ml) in the presence of DMSO, KYN (400 μM), or KYNA (200 μM) for 24 h. The expression of TSG-6 mRNA (left) and protein (right) were examined by real-time PCR and ELISA. **b** These cells (2.5 × 10^5^ cells per mouse) treated as in **a** for 48 h were intravenously injected into IBD mice on day 2. Changes in body weight during the entire experiment showed as the percentage of the initial body weight on day 0. **c** Colon length of IBD mice was assessed. **d** H&E staining of the colon sections and histological scores were shown. **e** IL-6 in the serum of IBD mice was analyzed by ELISA. Five mice per group were used. Scale bars, 100 μm. Results were shown as mean ± SEM. *P < 0.05, **P < 0.01, ***P < 0.001, ****P < 0.0001

We next tested whether the loss of the beneficial effects on IDO-KD-hMuSCs could be rescued by KYN or KYNA. Indeed, TSG-6 expression was largely restored in IDO-KD-hMuSCs treated with exogenous KYN or KYNA (Fig. 5a). We next examined whether KYN or KYNA could restore the beneficial effects of IDO-KD-hMuSCs on IBD mice. For this purpose, IDO-KD-hMuSCs were pretreated with KYN or KYNA and injected intravenously into IBD mice. As expected, parameters in body weight, colon length, bowel wall thickening, crypt damage, the infiltration of inflammatory cells in colons, and IL-6 level in serum were similar to those in the ctrl-hMuSC group (Fig. 5b–e). Collectively, these data demonstrated that hMuSCs ameliorate IBD via KYN or KYNA-mediated upregulation of TSG-6.

### KYN or KYNA promotes TSG-6 expression by activating AHR signaling pathway

**Fig. 6:**
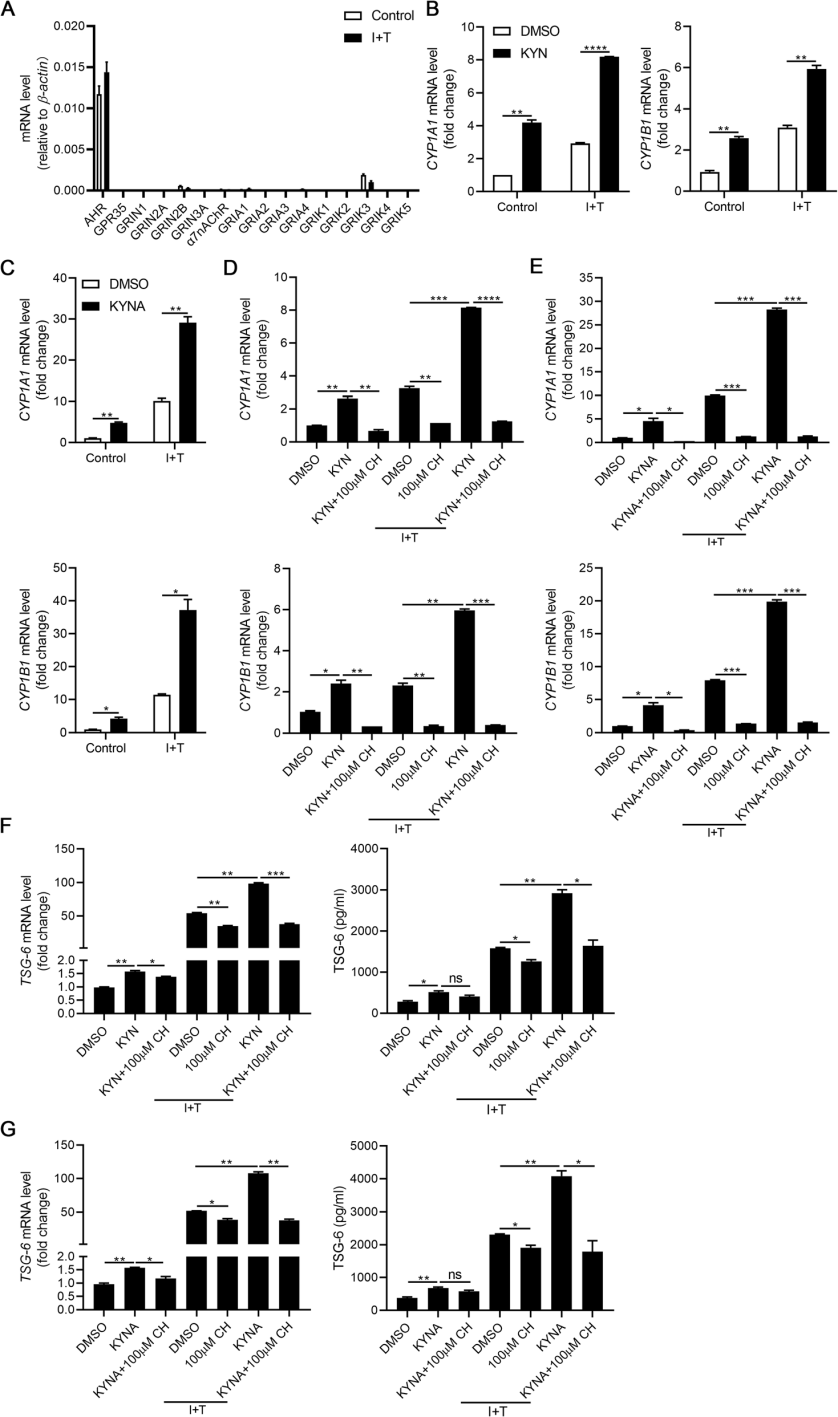
KYN or KYNA activates AHR signaling pathway to enhance TSG-6 production. **a** hMuSCs treated with IFN-γ (10 ng/ml) and TNF-α (10 ng/ml) were examined for the receptor of KYN and KYNA expression by real-time PCR. **b**, **c** hMuSCs were treated with IFN-γ (1 ng/ml) and TNF-α (10 ng/ml) in the presence of DMSO, KYN (400 μM), or KYNA (200 μM) for 24 h. The expression of *CYP1A1* and *CYP1B1* were examined by real-time PCR. **d**, **e** The expression of *CYP1A1* and *CYP1B1* in hMuSCs treated as in **b** in the presence or absence of CH-223191 for 24 h were examined by real-time PCR. **f**, **g** The expression of TSG-6 mRNA (left) and protein (right) were examined by real-time PCR and ELISA, respectively. Results were shown as mean ± SEM. *P < 0.05, **P < 0.01, ***P < 0.001; ns, no significance

KYNA was shown to promote the expression of TSG-6 through activating AHR signaling [16]. Besides, KYNA can also bind to various receptors to participate in immune regulation [34]. To investigate the detailed mechanism of KYN- and KYNA-regulated TSG-6 expression, their candidate receptors were examined. Among all these receptors, only AHR was found to be constitutively and highly expressed in hMuSCs (Fig. 6a). AHR, a ligand-activated transcriptional factor, belongs to the superfamily of basic helix-loop-helix/Per-ARNT-Sim (bHLH/PAS) [35]. AHR can recognize multitudinous ligands including main environmental pollutants, 6-formylindolo (3, 2-b) carbazole (FICZ), KYN, KYNA, xanthurenic acid, and cinnabarinic acid. After ligand binding, the cytoplasmic AHR translocates to the nucleus, dimerizes with AHR nuclear translocator (ARNT), and induces multiple AHR-responsive genes such as cytochrome P450 family 1A1 (*CYP1A1*) and cytochrome P450 family 1B1 (*CYP1B1*) [36, 37]. These effector genes participate in immune response, tumor promotion, redox regulation, detoxification, and cell division. Therefore, we next examined the nuclear translocation of AHR in hMuSCs treated with KYN or KYNA. We found that the expressions of *CYP1A1* and *CYP1B1*, which are transcriptional targets of AHR, were increased in hMuSCs treated with KYN or KYNA (Fig. 6b, c), indicating that KYN or KYNA can activate AHR signaling in hMuSCs. Furthermore, when CH-223191 was added to antagonize the nuclear translocation of AHR in hMuSCs, the inductions of CYP1A1 and CYP1B1 by KYN or KYNA were blocked (Fig. 6d, e). Moreover, CH-223191 also abolished the enhancement of TSG-6 expression caused by KYN or KYNA (Fig. 6f, g). Together, these results demonstrated that KYN or KYNA promotes TSG-6 production through activating their common receptor AHR.

## Discussion

**Fig. 7:**
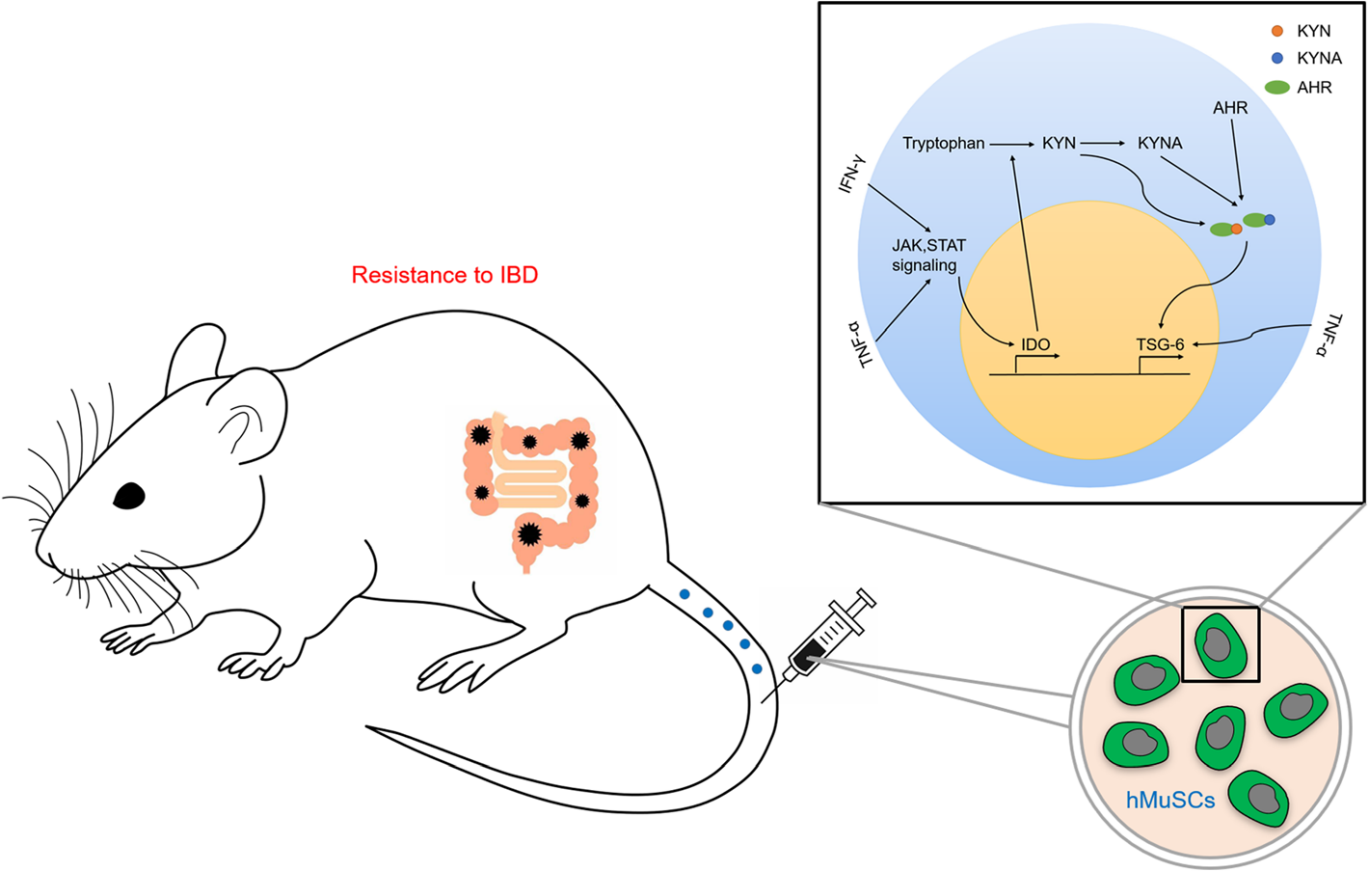
A schematic model of the anti-inflammatory effects of hMuSCs on DSS-induced IBD. IDO induced by IFN-γ and TNF-α catalyzes tryptophan metabolism to produce KYN or KYNA in hMuSCs. KYN or KYNA promotes TSG-6 production through activating AHR signaling pathway, and consequently alleviates IBD

As adult myogenic progenitor cells, MuSCs can maintain and restore muscle tissue through differentiating into myofibers. In addition to giving rise to myofibers, MuSCs have been shown to regulate collagen expression in fibrogenic cells via secreting exosomes containing miR-206, and change extracellular environments for muscle tissue maintenance and adaption [38], indicating that MuSCs can orchestrate tissue microenvironments through “cell empowerment.” Our previous study has demonstrated that mouse MuSCs have a therapeutic effect on IBD through producing IGF-2 to endow maturing macrophages anti-inflammatory properties [28]. Inflammatory stimuli can confer stem cells, particularly MSCs, a more potent ability to orchestrate tissue microenvironments [8, 11]. In this study, we demonstrated that hMuSCs treated with a combination of IFN-γ and TNF-α possess higher therapeutic efficacy on IBD than naïve hMuSCs, suggesting that tissue stem cells may generally exhibit an augmented immunomodulatory property in response to inflammatory cues. Furthermore, IDO-TSG-6 axis was found to mediate the anti-inflammatory effects of hMuSCs (Fig. 7).

As a metabolic enzyme, IDO is believed to contribute to immune homeostasis by tryptophan deprivation and production of diverse metabolites [39]. IDO inhibits T cell proliferation by triggering an amino acid-sensing signal-transduction pathway undergoing tryptophan depletion [14]. On the other hand, the IDO-mediated tryptophan catabolism plays a regulatory role in damping the activation of the immune system. Tryptophan metabolites through kynurenine pathway, such as KYN, KYNA, 3-hydroxyanthranilic acid, and quinolinic acid, have different effects on various immune cells, including T cells, B cells, NK cells, and antigen-presenting cells (APCs) [14, 15, 39]. The finding that hMuSCs exposed to the combined IFN-γ and TNF-α treatment can also acquire a potent anti-inflammatory property that is mediated by the IDO-TSG-6 axis indicates that the IDO-mediated immunoregulatory mechanism may operate in many types of cells.

We found that KYN or KYNA, two main tryptophan metabolites catalyzed by IDO, promoted TSG-6 production in hMuSCs. In vivo, exogenous KYN or KYNA can rescue the therapeutic defect of IDO-KD-hMuSCs in IBD mice by increasing TSG-6 expression. Both molecules have been reported to be capable of regulating immune responses. Specifically, KYN can suppress the activity of NK cells, DCs, and monocytes/macrophages [15]. Furthermore, KYN has been found to inhibit T cell proliferation and induce T cell death [40]. These mechanisms are partly mediated by activation of AHR [15]. Additionally, AHR activated by KYN can induce migration-associated gene expressions in cancer cells [41]. The KYN-AHR axis has been recognized as promising targets in inflammatory diseases and cancer therapy. KYNA shares the same receptor AHR with KYN. It has been demonstrated that KYNA binding to AHR induces IL-6 production in breast cancer cells [17]. However, KYNA has more potent binding capacity with AHR than KYN [17, 42]. It remains unclear whether KYN or KYNA can activate other immune pathways.

## Conclusion

In summary, our results show that hMuSCs licensed by inflammatory factors IFN-γ and TNF-α exhibit an anti-inflammatory function that depends on IDO-TSG-6 axis in DSS-induced IBD mice. Furthermore, the IDO metabolites KYN and KYNA directly regulate TSG-6 expression through activating AHR signaling. These results expand the potential applications of stem cell therapies in inflammatory diseases.

## Supplementary Information

**Additional file 1: Figure S1.** Phenotypic characterization of hMuSCs. **a, b** hMuSCs surface makers and nuclear factor PAX7 were characterized by flow cytometry analysis. **c** Representative images of the differentiation potentials of cultured hMuSCs. Red indicated myosin heavy chain (MyHC) staining. Hoechst indicated nuclei staining. Merge indicated merged images of MyHC and Hoechst staining. Scale bar, 50 μm. **Figure S2.** Inflammation-licensed hMuSCs possess more potent therapeutic efficacy on IBD. **a** Mice with DSS-induced IBD were administered intravenously with PBS, hMuSCs or hMuSCs pretreated with IFN-γ (10 ng/ml) and TNF-α (10 ng/ml) for 48 h (2.5 × 10^5^ cells) on day 2. Changes in body weight during the entire experiment were shown as the percentage of the initial body weight on day 0. **b** Colon length of IBD mice was assessed. **c** H&E staining of the colon sections and histological scores were shown. **d** IL-6 in the serum of IBD mice was analyzed by ELISA. Five mice per group were used. Scale bars, 100 μm. Results were shown as mean ± SEM. *P < 0.05, **P < 0.01, ***P < 0.001, ****P < 0.0001. **Figure S3.** The efficiency of IDO knockdown in hMuSCs. **a, b** Ctrl-hMuSCs and IDO-KD-hMuSCs were treated with IFN-γ (10 ng/ml) and TNF-α (10 ng/ml) for 24 h. The efficiency of IDO knockdown was measured by real-time PCR and Western blot. Results were shown as mean ± SEM. *P < 0.05, ***P < 0.001. **Figure S4.** The efficiency of TSG-6 knockdown in hMuSCs. **a, b** hMuSCs transfected with control siRNA (ctrl-siRNA-hMuSCs) or TSG-6 siRNA (TSG-6-siRNA-hMuSCs) were pretreated with IFN-γ (10 ng/ml) and TNF-α (10 ng/ml) for 24 h and 48 h to examine the expression of TSG-6 mRNA (left) and protein (right). **P < 0.01, ***P < 0.001. **Table S1.** The primers used for real-time PCR.

## Data Availability

The datasets used and/or analyzed during the current study are available from the corresponding author on reasonable request.

## Abbreviations

MuSCs: Muscle stem cells
MSCs: Mesenchymal stem cells
IFN-γ: Interferon-γ
TNF-α: Tumor necrosis factor-α
TSG-6: TNF-stimulated gene 6
IBD: Inflammatory bowel disease
DSS: Dextran sulfate sodium
ELISA: Enzyme-linked immunosorbent assay
IDO: Indoleamine 2,3-dioxygenase
KYN: Kynurenine
KYNA: Kynurenic acid
AHR: Aryl hydrocarbon receptor
IL-1β: Interleukin-1β
PGE_2_: Prostaglandin E2
TGF-β: Transforming growth factor-β
GAGs: Glycosaminoglycans
HA: Hyaluronan
HS: Heparan sulfate
IGF-2: Insulin-like growth factor-2
FBS: Fetal bovine serum
siRNAs: Small interfering RNAs
KAT: Kynurenine aminotransferases
HPLC: High-performance liquid chromatography
bHLH/PAS: Basic helix-loop-helix/Per-ARNT-Sim
FICZ: 6-Formylindolo (3, 2-b) carbazole
ARNT: AHR nuclear translocator
CYP1A1: Cytochrome P450 family 1A1
CYP1B1: Cytochrome P450 family 1B1
APCs: Antigen-presenting cells
