# Supplementary material for: Inflammatory cytokines-stimulated human muscle stem cells ameliorate ulcerative colitis via the IDO-TSG6 axis
Source: Stem Cell Res Ther. 2021 Jan 9;12:50. doi: 10.1186/s13287-020-02118-3 (PMC7796621; doi:10.1186/s13287-020-02118-3)
Supplement: Supplementary file 1 — Additional file 1: Figure S1. Phenotypic characterization of hMuSCs. a, b hMuSCs surface makers and nuclear factor PAX7 were characterized by flow cytometry analysis. c Representative images of the differentiation potentials of cultured hMuSCs. Red indicated myosin heavy chain (MyHC) staining. Hoechst indicated nuclei staining. Merge indicated merged images of MyHC and Hoechst staining. Scale bar, 50 μm. Figure S2. Inflammation-licensed hMuSCs possess more potent therapeutic efficacy on IBD. a Mice with DSS-induced IBD were administered intravenously with PBS, hMuSCs or hMuSCs pretreated with IFN-γ (10 ng/ml) and TNF-α (10 ng/ml) for 48 h (2.5 × 10^5^ cells) on day 2. Changes in body weight during the entire experiment were shown as the percentage of the initial body weight on day 0. b Colon length of IBD mice was assessed. c H&E staining of the colon sections and histological scores were shown. d IL-6 in the serum of IBD mice was analyzed by ELISA. Five mice per group were used. Scale bars, 100 μm. Results were shown as mean ± SEM. *P < 0.05, **P < 0.01, ***P < 0.001, ****P < 0.0001. Figure S3. The efficiency of IDO knockdown in hMuSCs. a, b Ctrl-hMuSCs and IDO-KD-hMuSCs were treated with IFN-γ (10 ng/ml) and TNF-α (10 ng/ml) for 24 h. The efficiency of IDO knockdown was measured by real-time PCR and Western blot. Results were shown as mean ± SEM. *P < 0.05, ***P < 0.001. Figure S4. The efficiency of TSG-6 knockdown in hMuSCs. a, b hMuSCs transfected with control siRNA (ctrl-siRNA-hMuSCs) or TSG-6 siRNA (TSG-6-siRNA-hMuSCs) were pretreated with IFN-γ (10 ng/ml) and TNF-α (10 ng/ml) for 24 h and 48 h to examine the expression of TSG-6 mRNA (left) and protein (right). **P < 0.01, ***P < 0.001. Table S1. The primers used for real-time PCR. [file 13287_2020_2118_MOESM1_ESM.pdf]

SUPPLEMENTARY FIGURE

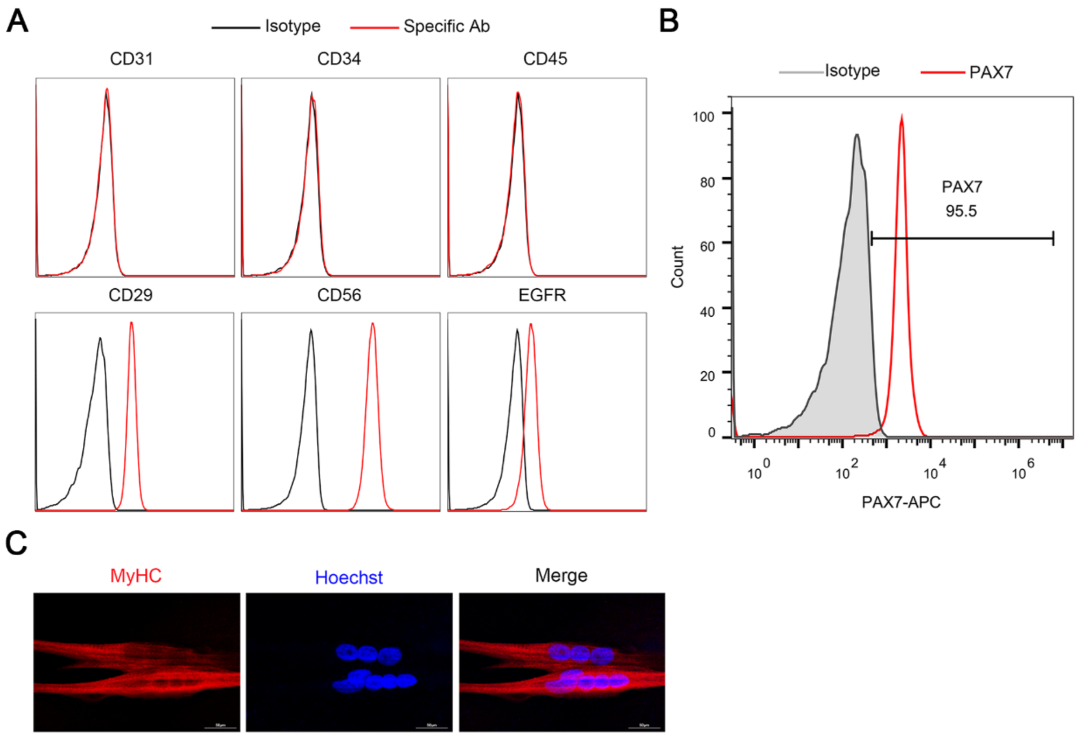

Figure S1 Phenotypic characterization of hMuSCs

a, b hMuSCs surface makers and nuclear factor PAX7 were characterized by cytometry analysis. **c** Representative images of the differentiation potentials of cultured hMuSCs. Red indicated myosin heavy chain (MyHC) staining. Hoechst indicated nuclei staining. Merge indicated merged images of MyHC and Hoechst staining. Scale bar, 50 µm.

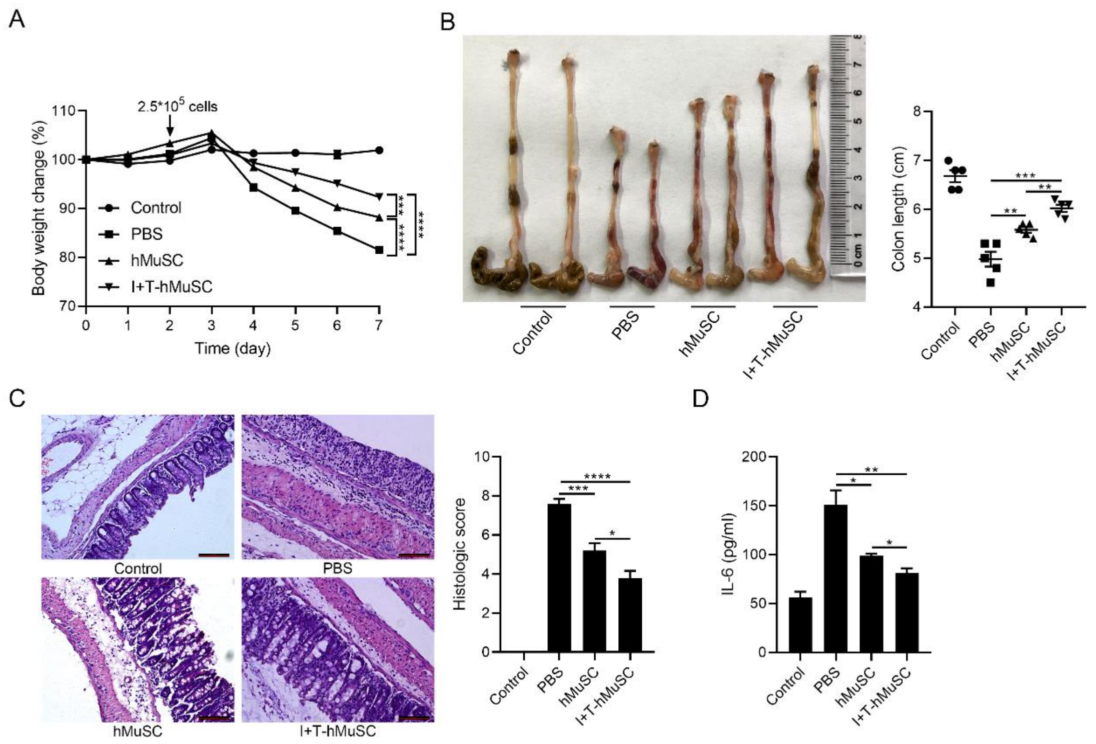

Figure S2 Inflammation-licensed hMuSCs possess more potent therapeutic efficacy on IBD

a Mice with DSS-induced IBD were administered intravenously with PBS, hMuSCs or hMuSCs pretreated with IFN-γ (10 ng/ml) and TNF-α (10 ng/ml) for 48 h (2.5×10^5 cells) on day 2. Changes in body weight during the entire experiment were shown as the percentage of the initial body weight on day 0. **b** Colon length of IBD mice was assessed. **c** H&E staining of the colon sections and histological scores were shown. **d** IL-6 in the serum of IBD mice was analyzed by ELISA. Five mice per group were used. Scale bars, 100 μm. Results were shown as mean ± SEM. *$P < 0.05$, **$P < 0.01$, ***$P < 0.001$, ****$P < 0.0001$.

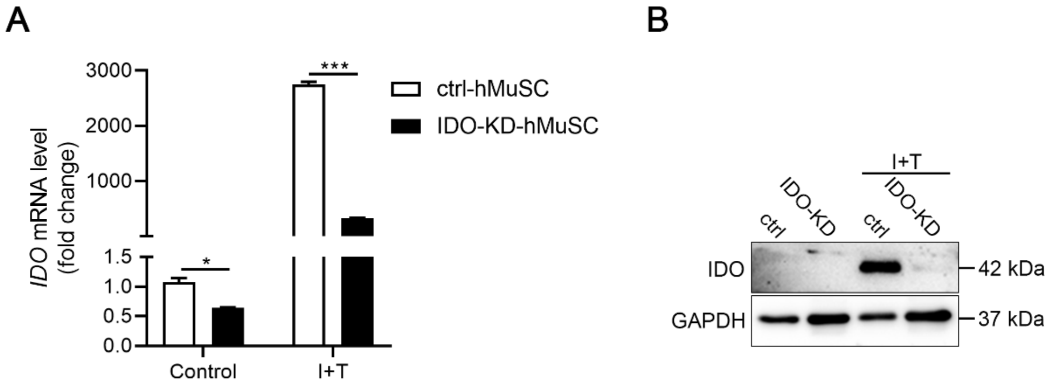

Figure S3 The efficiency of IDO knockdown in hMuSCs

a, b Ctrl-hMuSCs and IDO-KD-hMuSCs were treated with IFN-γ (10 ng/ml) and TNF-α (10 ng/ml) for 24 h. The efficiency of IDO knockdown was measured by real-time PCR and Western blot. Results were shown as mean ± SEM. *$P < 0.05$, ***$P < 0.001$.

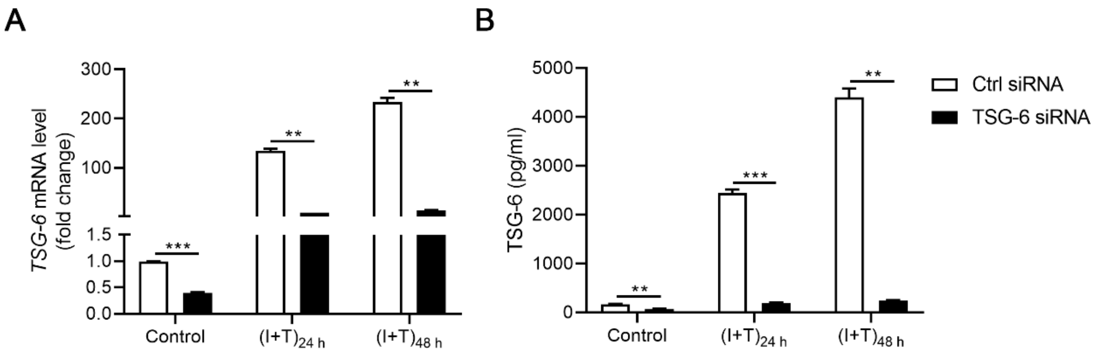

Figure S4 The efficiency of TSG-6 knockdown in hMuSCs

a, b hMuSCs transfected with control siRNA (ctrl-siRNA-hMuSCs) or TSG-6 siRNA (TSG-6-siRNA-hMuSCs) were pretreated with IFN-γ (10 ng/ml) and TNF-α (10 ng/ml) for 24 h and 48 h to examine the expression of TSG-6 mRNA (left) and protein (right). $^{**}P < 0.01$, $^{***}P < 0.001$.

SUPPLEMENTAL TABLE

Table 1 The primers used for real-time PCR

Genes name (Human)	Oligonucleotide sequence (5′-3′)
β-actin	F: TTGCCGACAGGATGCAGAAGGA
	R: AGGTGGACAGCGAGGCCAGGAT
AHR	F: ATTGTGCCGAGTCCCATATC
	R: AAGCAGGCGTGCATTAGACT
CYP1A1	F: CTTGGACCTCTTTGGAGCT
	R: GACCTGCCAATCACTGTG
CYP1B1	F: GCAAGGGCATGGGAATTGAC
	R: GAGTCTCTTGGCGTCGTCAG
GRIA1	F: TGCTTTGTCGCAACTCACAGA
	R: GGCATAGACTCCTTTGGAGAAC
GRIA2	F: TGCATACCTCTATGACAGTGACA
	R；AGAATTACACGCCGTTCCTTT
GRIA3	F: ACCATCAGCATAGGTGGACTT
	R: GGTTGGTGTTGTATAACTGCACG
GRIA4	F: CAGCGCCTTACATATCTCCCT
	R: GAAGGTCTTAGTTGCAGCACA
GRIK1	F: TGCTCTCGAAGTTCCACACAT
	R: CCTGTGCTGTCTTCATACACC
GRIK2	F: CCTGTGCTGTCTTCATACACC

	R: GCTCCCATTGGGCCAGATT
GRIK3	F: TTCGAGGCGACCAAAAAGG
	R: GGTTCACGTAGAAGGTGTCCT
GRIK4	F: CAAGGCCAAGGTCGAAGTG
	R: CTCCACAGATGTTGCTGATGAT
GRIK5	F: CCACCGTGAGCCATATCTGTG
	R: CGCGAAGCGAAGGTACTGAA
GRIN1	F: ACCCCAAGATCGTCAACATTG
	R: GGCTAACTAGGATGGCGTAGA
GRIN2A	F: TCATGCAGGATTATGACTGGCA
	R: TGTGGTCTTGACGAAGCTGAT
GRIN2B	F: TCTGACCGGAAGATCCAGGG
	R: TCCATGATGTTGAGCATTACGG
GRIN3A	F: CGGGACGCCCTCCTATTTG
	R: CCACGGTATGGCACACACT
GRP35	F: CTCCCTGCGAGACACCTCA
	R: CTGATGCTCATGTACCTGTTGG
IDO	F: GCCCTTCAAGTGTTTCACCAA
	R: CCAGCCAGACAAATATATGCGA
KAT I	F: GTCGTCCTGTGTTTGTGTCCCT
	R: GCTTTGGTGCGTGATGTGAA
KAT II	F: GCGGCGAGTCCTCAAC

	R: ACCAGCCAAGGAGATCATCG
KAT III	F: CCTCAAGCCCAGTCGGGA
	R:TCAGTGACATTTTAGCAGAAGTAGA
KAT IV	F: TGCGAAAACAATGGCTGCAA
	R: TGCGAAAACAATGGCTGCAA
KMO	F: TGCGAGCACATGTCAACTCAA
	R: TTTGCCAATGCCAACGCT
KYNU	F: GGCTCTCCACCTAGATGAGGA
	R: TCTCTAAAGCTCTTGTCCTTGACT
TSG-6	F: TGTCTGTGCTGCTGGATGGAT
	R: TGTGGGTTGTAGCAATAGGCAT
3-HAO	F: CCAGGAAGGACTATCACATCGA
	R: TATCTCTCCCTGCCGAATGAC
7nAChR	F: GCTGGTCAAGAACTACAATCCC
	R: CTCATCCACGTCCATGATCTG
